# Clotrimazole Fluidizes Phospholipid Membranes and Localizes at the Hydrophobic Part near the Polar Part of the Membrane

**DOI:** 10.3390/biom11091304

**Published:** 2021-09-02

**Authors:** Alessio Ausili, Illya Yakymenko, José A. Teruel, Juan C. Gómez-Fernández

**Affiliations:** Department of Biochemistry and Molecular Biology (A), Faculty of Veterinary Science, International Campus of Excellence Mare Nostrum, Universidad de Murcia, Apartado. 4021, E-30100 Murcia, Spain; aausili@um.es (A.A.); illya.yakymenko@um.es (I.Y.); teruel@um.es (J.A.T.)

**Keywords:** clotrimazole, model membrane, location, membrane fluidity

## Abstract

Clotrimazole (1-[(2-chlorophenyl)-diphenylmethyl]-imidazole) is an azole antifungal drug belonging to the imidazole subclass that is widely used in pharmacology and that can be incorporated in membranes. We studied its interaction with 1,2-dimyristoyl-sn-glycero-3-phosphocholine (DMPC) phospholipid vesicles by using differential scanning calorimetry and found that the transition temperature decreases progressively as the concentration of clotrimazole increases. However, the temperature of completion of the transition remained constant despite the increase of clotrimazole concentration, suggesting the formation of fluid immiscibility. ^1^H-NMR and ^1^H NOESY MAS-NMR were employed to investigate the location of clotrimazole in 1-palmitoyl-2-oleoyl-sn-glycero-3-phosphocholine (POPC) phospholipid membranes. In the presence of clotrimazole, all the resonances originating from POPC were shifted upfield, but mainly those corresponding to C2 and C3 of the fatty acyl, chains suggesting that clotrimazole aromatic rings preferentially locate near these carbons. In the same way, 2D-NOESY measurements showed that the highest cross-relaxation rates between protons of clotrimazole and POPC were with those bound to the C2 and C3 carbons of the fatty acyl chains. Molecular dynamics simulations indicated that clotrimazole is located near the top of the hydrocarbon-chain phase, with the nitrogen atoms of the imidazole ring of clotrimazole being closest to the polar group of the carbonyl moiety. These results are in close agreement with the NMR and the conclusion is that clotrimazole is located near the water–lipid interface and in the upper part of the hydrophobic bilayer.

## 1. Introduction

**Figure 1:**
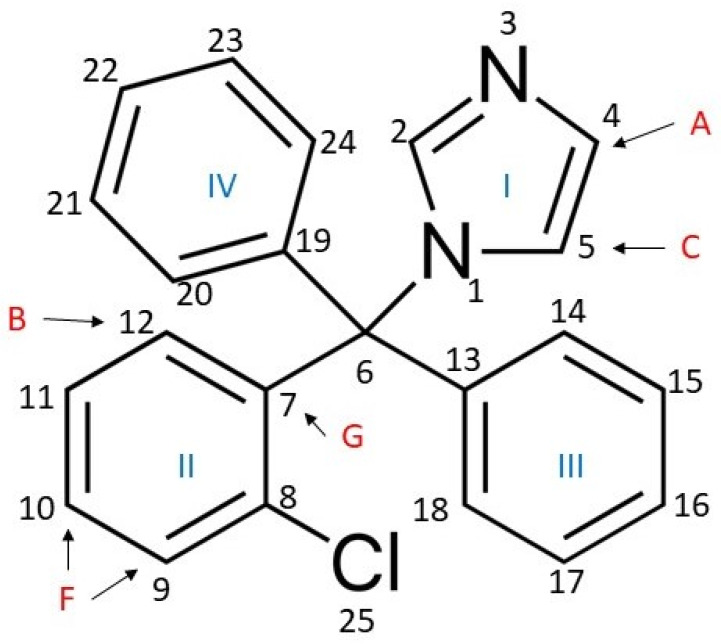
Structure of clotrimazole. Protons studied by ^1^H-NMR are symbolised as follows, according to the carbon to which they are linked: atom 4, A; atom 12, B; atom 5, C; atoms 5, 14, 18, 20 and 24, D; atoms 11, 15, 16, 17, 21, 22 and 23, E; atoms 9 and 10, F; atom 2, G.

Clotrimazole (1-[(2-chlorophenyl)-diphenylmethyl]-imidazole) is an azole antifungal drug belonging to the imidazole subclass with a molecular weight of 344.8 g/mol. The clotrimazole molecule consists of a quaternary carbon substituted with an imidazole group, two phenyl rings and a phenyl ring with a chloro-substitution at the ortho-position. Its spatial conformation is tetrahedral (Figure 1).

Its principal medicinal use is for the treatment of vaginal and oral candidiasis [1,2] and athlete’s foot [3], caused by *Candida albicans* and different fungi, respectively, although it is also used for infections caused by other fungi. Its mechanism of action involves the inhibition of Cyp51p (cytochrome P450 14alpha-demethylase), which causes the demethylation of 14-α-lanosterol, Cyp51p. This enzyme is involved in the synthesis of ergosterol, which is the major component of fungal cytoplasmic membranes. Blocking the ergosterol synthesis pathway leads to the accumulation of 14-α-methylated sterols, resulting in a structural and functional defect of the membrane [4].

In addition to effects on Cyp51p, an influence on Ca^2+^-related metabolism has been observed. These effects include inhibition of sarcoplasmic reticulum Ca^2+^-ATPase (SERCA) [5], gastric H^+^-K^+^-ATPase [6] and Na^+^-K^+^-ATPase [7], but it is not known if it has a specific binding site on these proteins, so its mechanism of action could be based on its perturbation of membrane structure and dynamics.

It is also worthwhile mentioning that clotrimazole has been associated with lipid particles for pharmacological applications. Clotrimazole is orally administered for the treatment of systemic candidiasis (pulmonary and disseminated cryptococcosis and aspergillosis). However, because of its adverse effects following systemic administration, it is mainly used for the treatment of localized topical candidiasis. Further, its topical effectiveness seems to be strongly affected by the formulation [8,9], which may play a key role in ensuring the permanence and promoting the penetration of drugs in the skin and biofilm’s matrix [10]. To reach this goal, over the past few decades new topical delivery systems have been explored [11,12]. In particular, it has recently been shown that liposomal formulations in the form of three-dimensionally structured hybrid vesicles clearly improve the cutaneous delivery of clotrimazole for the treatment of topical candidiasis [13]. Given the hydrophobic nature of clotrimazole and its capacity to insert into membranes, it would be interesting to understand its interaction with membranes and its location within phospholipid bilayers. In this study, we used 1,2-dimyristoyl-sn-glycero-3-phosphocholine (DMPC) and 1-palmitoyl-2-oleoyl-sn-glycero-3-phosphocholine (POPC) vesicles. DMPC was used for the differential scanning calorimetry studies. This phospholipid is one of the most commonly used phospholipids for this purpose. POPC was used for the ^1^H-NMR studies. POPC is widely used because it is considered a model of the most common phospholipids in animal tissues. However, since its transition temperature is below zero, DMPC, with a transition temperature at 23.5 °C, is preferred. Nevertheless, an important advantage of using POPC for ^1^H-NMR studies is that the double bond of the oleoyl residue provides a resonance that serves as a reference at the middle of the monolayer due to the protons related to the double bond. After using differential scanning calorimetry (DSC), ^1^H-NMR and ^1^H NOESY MAS-NMR to locate the clotrimazole molecule in POPC vesicles, we determined that it could be mainly found in the part of the hydrophobic bilayer that it is close to the lipid–water interface. These results are in very good agreement with studies using molecular dynamics simulations

## 2. Materials and Methods

### 2.1. Materials

1,2-Dimyristoyl-sn-glycero-3-phosphocholine (DMPC) and 1-palmitoyl-2-oleoyl-sn-glycero-3-phosphocholine (POPC) were purchased from Avanti Polar Lipids (Alabaster, AL, USA). Clotrimazole, 2H2O (99%) and all other reagents and solvents used in the experiments were acquired from Sigma-Aldrich (Madrid, Spain)

### 2.2. Sample Preparation

DMPC or POPC dispersions in the presence of different concentrations of clotrimazole were analysed by differential scanning calorimetry (DSC) and ^1^H NOESY MAS-NMR, respectively. The sample preparations of both experimental techniques were essentially identical, except for the type and amount of phosphatidylcholine, the lipid/clotrimazole molar ratio and the solvent used in the preparation of the buffers, being H2O for DSC and 2H2O for NMR.

Typically, the desired amounts of phosphocholine (PC) and clotrimazole, both dissolved in chloroform/methanol solution (2:1), were mixed in appropriate laboratory tubes. The solvents were then evaporated by a flow of nitrogen and then by high vacuum for at least 3 h to remove any residual solvent. The dried samples were then hydrated with 10 mM Hepes pH 7.4 and vortexed, at a temperature above the phase transition temperature of the phospholipid, to form a homogeneous suspension of multilamellar vesicles (MLVs).

The concentration of the phospholipids was determined by the Böttcher method [14].

### 2.3. Differential Scanning Calorimetry

Samples for analysis by DSC were prepared at a DMPC concentration of 1.36 mM and the DMPC/clotrimazole molar ratios were 50:1, 20:1, 10:1, 5:1 and 2:1; a sample of pure DMPC was also used.

Measurements were recorded with a MicroCal MC-2 microcalorimeter (Microcal, Northampton, MA, USA) using the same buffer in the reference cell as used to prepare the MLVs. Before loading, the samples and reference were degassed for 10 min under vacuum. Three thermograms were subsequently recorded from 10 to 40 °C with a scan rate of 1 °C/min, and the last scan was used for the data processing. A further buffer vs. buffer scan was recorded for subtraction from each of the sample thermograms before analysis. The data analysis was performed with OriginPro 8.5 software (OriginLab Corporation, Northampton, MA, USA), and the parameters calculated from the final thermograms were the transition temperatures and enthalpies. The phase transition temperature corresponded to the maximum peak height associated with it, while the start and end of the transition were considered to be the temperature values corresponding to 5% of the peak height.

### 2.4. ^1^H NOESY MAS-NMR

A suspension of multilamellar vesicles (MLVs) was prepared in deuterated water containing 23.6 mmol of POPC and the corresponding amount of clotrimazole to obtain a POPC/clotrimazole molar ratio of 5:1. To obtain the MLVs, we proceeded as described above, hydrating the sample with 50 μL of buffer in 10 μL intervals and heating at 40 °C to facilitate clotrimazole incorporation into the vesicles. Finally, the obtained samples were inserted into a 4 mm MAS rotor.

NMR experiments were performed on a Bruker Avance 600 spectrometer (Bruker, Ettlingen, Germany) operating at 600 MHz and equipped with a HR-MAS probe and a 4 mm ZrO2 BL4 rotor with Kel-f BL4 cap. Measurements were carried out at a temperature of 25 °C. The spin rate was 8 kHz, obtaining 1024 data points across 16 scans, and the spectral width was 20 ppm. The relaxation time was 3.5 s and the mixing time was 300 ms. The 2D-NOESY experiments were performed using 90° pulses of 5.5 μs. The data obtained were analysed using TopSpin 3.5 software, supplied by Bruker. The rates of cross-relaxation velocities were quantified by means of the following equation [15]:

(1) $$\sigma_{ij}=\;\frac{A_{ij}\left(t_{m}\right)}{A_{jj}\left(t_{m}\right)\cdot t_{m}}$$

where *σ_ij_* is the cross-relaxation rate, *A_ij_* is the cross-peak volume, *A_jj_* is the diagonal peak volume and *t_m_* is the mixing time (300 ms).

### 2.5. Molecular Dynamics Simulations

The molecular structure of clotrimazole was obtained from the PubChem Substance and Compound database [16] through the unique chemical structure identifier CID 2812. Molecular dynamics simulations were done with GROMACS software, version 5.0.7 and 2018.1 [17]. In this work, the united-atom model described by the Gromos 53A6 force field was used to build all molecules [18,19], since this force field has been proven to reproduce the experimental data for lipid membrane systems [20]. The topology file for POPC was used as described by Poger et al. [21,22], while the topology file for clotrimazole was obtained using the Automated Topology Builder and Repository [23,24]. Each membrane leaflet, containing 64 POPC molecules and 13 clotrimazole molecules, was hydrated with 2500 molecules of water (SPC model). The hydrated membrane bilayer was built with Packmol software [25]. Other conditions were used as previously described [26,27]. The last 60 ns from the trajectory of the production run were selected for analysis purpose using GROMACS analysis tools. Molecular dynamics calculations were carried out in the Computational Service of the University of Murcia (Spain).

## 3. Results

### 3.1. Differential Scanning Calorimetry Studies of the Interaction of Clotrimazole with DMPC Membranes

**Figure 2:**
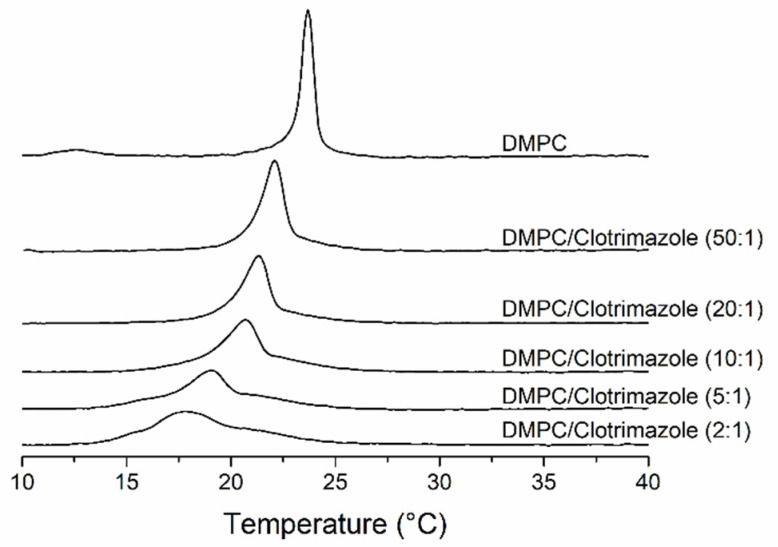
Thermograms of mixtures of 1,2-dimyristoyl-sn-glycero-3-phosphocholine (DMPC) with clotrimazole obtained by differential scanning calorimetry. The clotrimazole concentration (as a molar ratio) is shown beside each curve. The thermograms are normalised for the same amount of lipid.

Differential scanning calorimetry (DSC) is a very useful technique for the thermodynamic characterization of phospholipid systems. Using this method, it is possible to analyse both membrane phase transitions and how the presence of certain compounds incorporated into the membranes, as happens with clotrimazole, influence these parameters. Figure 2 shows thermograms of DMPC in the absence and presence of different concentrations of clotrimazole, displayed by the values of the heat capacity at constant pressure (Cp) as a function of temperature (from 10 to 40 °C). Visually, it is possible to observe a considerable influence of clotrimazole both on the pre-transition, indicative of the transition from the gel (L_β’_) to ripple (P_β’_) phase, and on the gel to liquid-crystalline phase transition of DMPC. Even at the lowest concentration of clotrimazole used (DMPC/clotrimazole 50:1 molar ratio), the total disappearance of the DMPC pre-transition can be seen around 12.5 °C, which is a typical effect due to intrinsic molecules, while the main transition temperature underwent an evident decrease from 23.7 to 22.1 °C. This temperature decreased progressively as the concentration of clotrimazole increased, until it reached the lowest temperature, which was recorded at the lipid/clotrimazole 2:1 molar ratio, of 17.8 °C.

Unlike the effect reported on the gel to liquid-crystalline transition temperature, clotrimazole did not seem to particularly influence the enthalpy associated with the process. As can be seen in Table 1, the ΔH values were basically identical or changed insignificantly for all the samples analysed.

**Figure 3:**
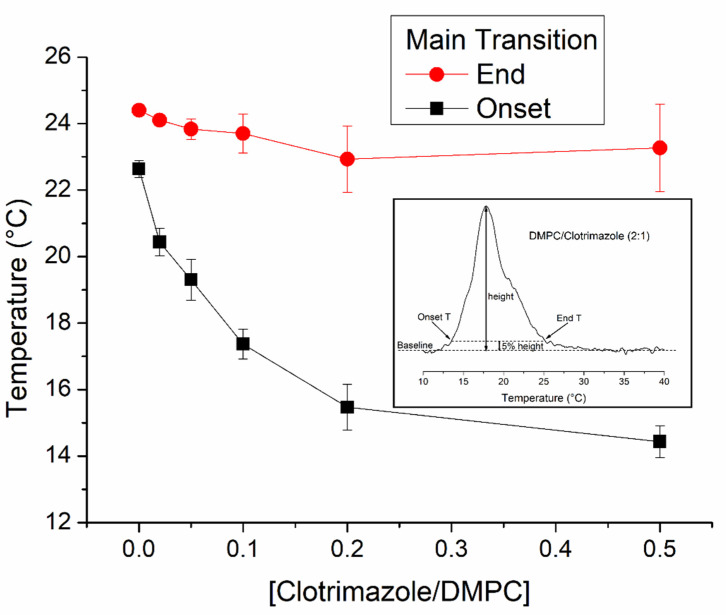
Partial phase diagram for DMPC in mixtures with clotrimazole. The black squares and red circles correspond to the onset and end temperatures of the main phase transitions and represent the solidus and fluidus lines, respectively. The standard deviation is represented in the graph by error bars. The inset of the figure shows, as an example, how the onset and end temperatures of the DMPC/clotrimazole 2:1 molar ratio sample were calculated. For all samples, the onset and end temperatures were assumed to be the temperatures corresponding to 5% of the maximum peak height. In this way, it was possible to determine these temperatures independently of the transition peak shape.

Using the temperatures derived from the DSC thermograms shown in Figure 2, a partial phase diagram was constructed for the phospholipid component (Figure 3) [28]. The diagram shows the onset and end temperatures of the gel to liquid-crystalline transition of DMPC as a function of the clotrimazole/lipid molar ratio. This generates two lines called solidus and fluidus lines; the first one is due to the temperatures of the onset of the transition and the second one the end temperatures. This diagram shows that, by increasing clotrimazole concentration, the fluidus line remains constant in a near-ideal manner. This indicates a fluid immiscibility that can be attributed to the formation of clotrimazole aggregates. In contrast, the solidus line gradually decreases from 22.6 ± 0.2 to 14.4 ± 0.5 °C with the 2:1 DMPC/clotrimazole molar ratio.

### 3.2. ^1^H-NMR and ^1^H NOESY MAS-NMR Results Indicated That Clotrimazole Is Located near the Water–Lipid Interface and Located in the Upper Part of the Hydrophobic Bilayer

We used POPC for this study because it is a very common component of biological membranes and it forms fluid membranes at 25 °C. Furthermore, POPC is very useful for these types of studies using 2D-^1^H-NMR NOESY because it has a double bond in the oleoyl chain and the resonances given by the protons related to this double bond provide a reference situated between the C3 carbon and the terminal methyl of the fatty acyl chain.

We employed ^1^H-NMR-MAS to study the location of clotrimazole in POPC membranes. Figure S1 (Supplementary Materials) shows the ^1^H-NMR-MAS 1D spectra of the POPC bilayers to which clotrimazole was incorporated at a 5:1 POPC-to-clotrimazole molar ratio.

**Figure 4:**
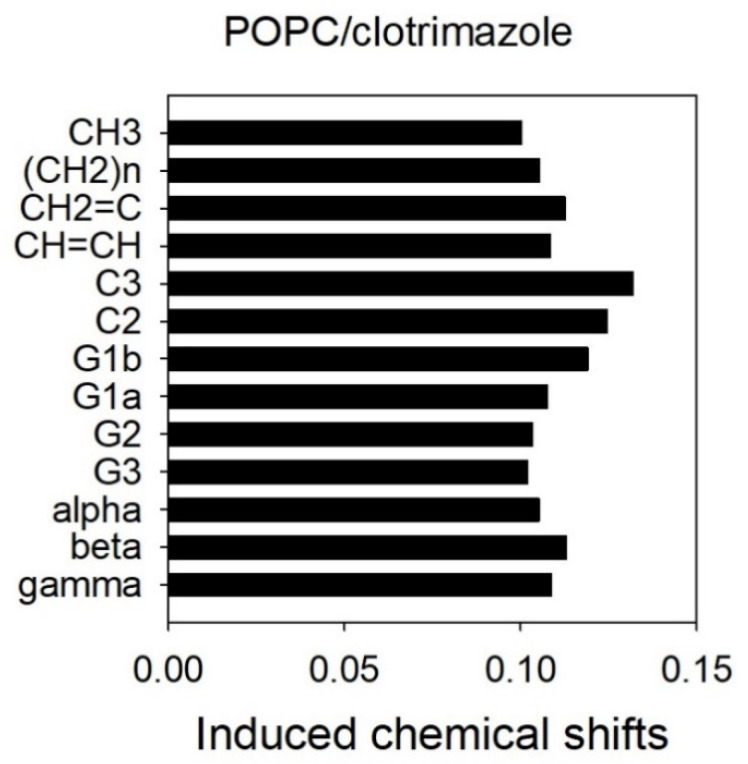
Induced chemical shifts observed in the resonances of the protons of 1-palmitoyl-2-oleoyl-sn-glycero-3-phosphocholine (POPC) along the long axis of the molecule from the centre of the membrane to the polar group after the incorporation of clotrimazole. The shifts were calculated by subtracting the ^1^H-NMR chemical shifts observed in the presence of clotrimazole from those of the pure POPC.

In the presence of clotrimazole, all the resonances originating from POPC were shifted upfield (Figure 4). These shifts are supposed to originate from the interaction of the ring current of the aromatic groups of clotrimazole with the protons of POPC, and larger shifts may be associated with greater proximity to the aromatic group. As shown in Figure 4, the largest shifts were seen for protons bound to C3 and C2 of the fatty acyl chains. It can be concluded that the aromatic rings are close to the first carbons of the fatty acyl chains and thus not far away from the polar groups of the phospholipids.

To further investigate the location of clotrimazole, we used 2D-NOESY measurements to determine the correlation between given protons of this molecule, which are labelled in Figure 1, and protons bound to POPC through the measurement of the cross-peaks.

**Figure 5:**
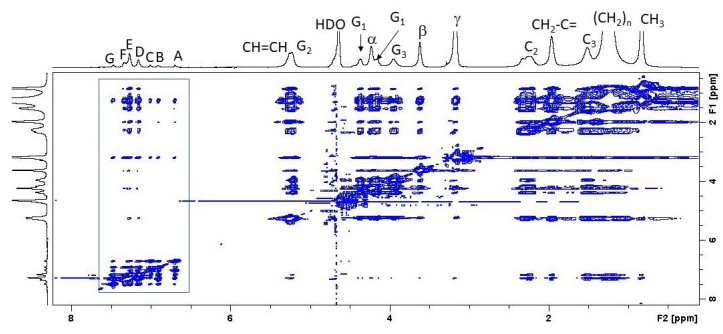
^1^H NOESY MAS-NMR spectrum of a POPC/clotrimazole sample. The molar ratio was 5:1 phospholipid/clotrimazole and the temperature was 25 °C. The spectrum was obtained at a mixing time of 300 ms. A, B, C, D, E, F and G are used to designate the protons bound to carbons of clotrimazole, as shown in Figure 1. The studied cross-peaks are within the framing.

Figure 5 depicts the 2D-NOESY spectrum of the POPC/clotrimazole spectrum. Clotrimazole shows seven resonances that are within the framing drawn in Figure 5 and that are clearly different from those corresponding to the phospholipids. These resonances are called as in Figure 1. These groups show cross-peaks with most phospholipid groups, although of very different sizes.

Cross-peaks related to groups A, B, C, D, E, F and G of clotrimazole are labelled in Figure S1 (Supplementary Materials). The closeness of the clotrimazole protons with the protons of POPC can be deduced by measuring the cross-peaks’ volumes, and the relative location of clotrimazole with respect to POPC can be determined from a quantitative analysis of cross-relaxation rates [29].

In accordance with the extension of the cross-relaxation rates between protons of clotrimazole and POPC, it is possible to estimate the probability of proximity between these protons, and this probability becomes higher as the rates are become larger.

**Figure 6:**
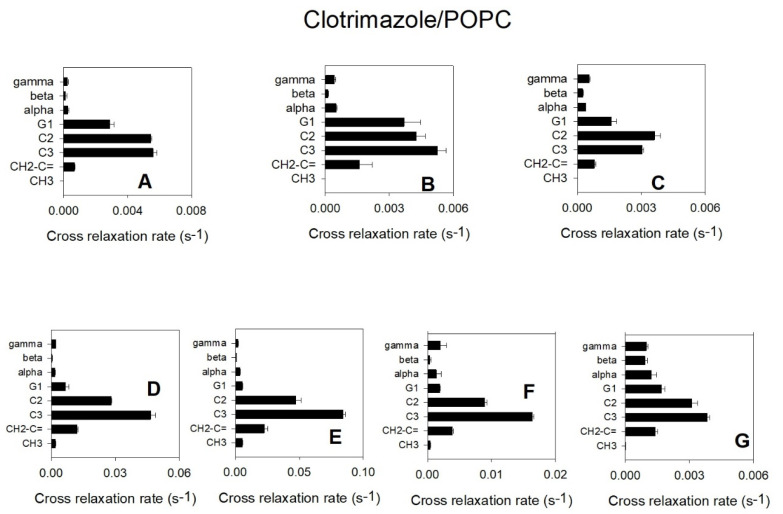
Cross-relaxation rates obtained from the ^1^H-NMR NOESY spectrum of POPC/clotrimazole. Cross-relaxation rates correspond to the protons bound to the different POPC groups along the long axis of the molecule from the polar group to the centre of the membrane (shown in ordinates) with respect to the clotrimazole carbons. Mean values ± standard deviations (five determinations). A, B, C, D, E, F and G are used to designate the protons bound to carbons of clotrimazole.

Figure 6 depicts a correlation between the different POPC groups represented in the ordinate axis ordered according to their location, from the most polar to the one located closest to the centre of the bilayer. It can be observed that the largest correlation rates were those corresponding to C3 and C2 for all the clotrimazole protons, indicating that this molecule is mainly located in the hydrophobic part of the membrane that is close to the lipid–water interface. The clotrimazole molecule is tetrahedral with the four cycles occupying the four vertexes. It can be deduced from Figure 5 that both proton C and, less so, proton A are bound to cycle I and occupy a more polar position than the other protons, since they are closer to C2. Cycle I is the most polar of the four due to its imidazole structure. Protons B, F and G are bound to cycle II and they are closer to C3 than to C2. A similar case is that of the protons grouped under D, which are bound to cycles III and IV. A similar situation can also be observed for the protons grouped under E, which are bound to cycles II, III and IV.

We can conclude that the main location of clotrimazole is in the upper part of the fatty acyl palisade, close to the C2–C3 carbons of these fatty acyl chains and not far away from the lipid–water interface.

### 3.3. Molecular Dynamics Simulations

In this work, ^1^H-NMR and ^1^H NOESY MAS-NMR techniques were used to experimentally probe the location of clotrimazole in a POPC bilayer. Additionally, we took advantage of molecular dynamics simulations to support the experimental results from NMR and to obtain more detailed information at the atomic level on the location of clotrimazole in the POPC bilayer.

**Figure 7:**
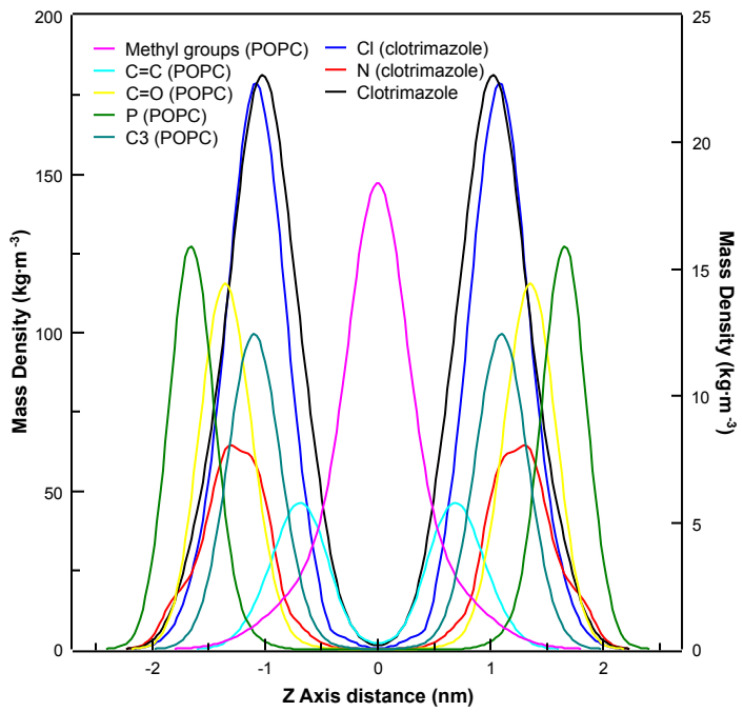
Mass density profiles along the z-axis of the membrane (normal to the bilayer) are shown for the methyl terminals of POPC (pink), the POPC double bond (cyan), the carbonyl groups of POPC (yellow), the P atom of POPC (green), the C3 atoms of POPC (teal), the chlorine atom of clotrimazole (blue), the nitrogen atom of clotrimazole (red) and the clotrimazole molecule (black). All curves correspond to the left-axis scale, except the chlorine and nitrogen atoms of clotrimazole which correspond to the right-axis scale.

Figure 7 shows the averaged mass density along the direction normal to the membrane surface (*z*-axis). The phosphorus atoms of POPC are in the outermost of the density profile figures (green), indicating the polar headgroup region of POPC; meanwhile the methyl terminal groups of POPC are located in the centre of the membrane (pink). The clotrimazole molecule (black) can be found between the carbonyl groups or C1 atoms of POPC (yellow) and the double bond of POPC (cyan), which corresponds to the C9 atom but centred nearest the C3 atom of POPC (teal). The chlorine atom of clotrimazole (blue) is also centred about the C3 atom, slightly towards the carbonyl groups. However, the nitrogen atoms of clotrimazole (red) are clearly located about the level of the carbonyl groups’ z-axis. These results are essentially the same as those shown above for the chemical shifts (Figure 4) and the cross-relaxation rates (Figure 6), indicating that clotrimazole is located near the top of the hydrocarbon-chain phase, the nitrogen atoms of the imidazole ring of clotrimazole being closest to the polar group of the carbonyl moiety.

**Figure 8:**
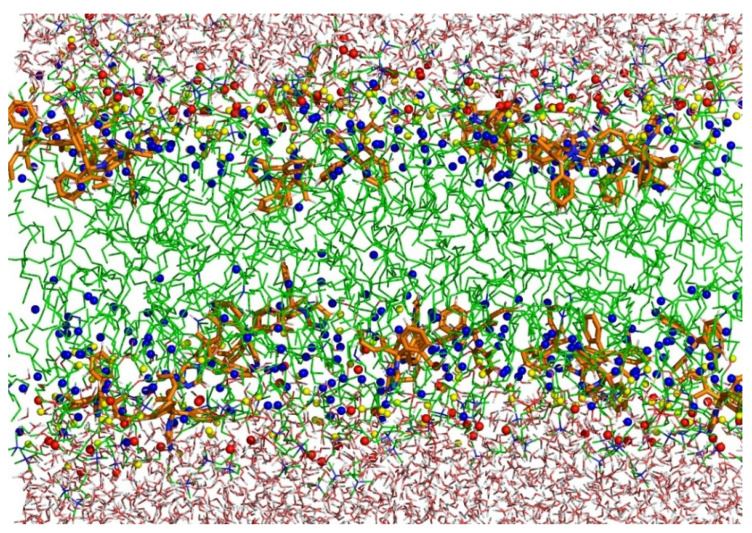
Representative snapshot of POPC bilayer with clotrimazole. POPC and water molecules are depicted as sticks. The POPC carbon backbone is depicted as green sticks, and the clotrimazole carbon backbone as orange sticks. Oxygen atoms are shown in red and hydrogen atoms in white. C3 atoms of POPC are shown as blue spheres, phosphorus atoms as red spheres and carbonyl groups of POPC as yellow spheres.

A representative snapshot of the POPC bilayer containing clotrimazole molecules is shown in Figure 8, where P, C=O and C3 atoms have been labelled for clarity. As described above (Figure 4 and Figure 5), clotrimazole molecules are found mainly close to the polar interface of the bilayer, in the vicinity of the carbonyl groups and C2–C3 atoms of POPC.

Despite the hydrophobicity conferred by the presence of three phenyl rings to the clotrimazole molecule, the tetrahedral arrangement of the substituents and the presence of a chlorine atom, and especially of an imidazole ring, could explain why the clotrimazole molecule is found in the most hydrophilic part of the hydrocarbon chains of the bilayer, while its presence is not observed in the centre of the membrane.

## 4. Discussion

Clotrimazol is a lipophilic compound used as an antifungal drug that is incorporated into membranes, where it may interfere with the activity of membrane enzymes, such as SERCA ATPases [5,6,7]. However, the mechanism of action of clotrimazole in acting on these enzymes is not clear. It seems to affect the binding of Ca^2+^, favouring E2 conformation in the case of the sarcoplasmic reticulum ATPase [5]. In the cases of both the gastric H^+^-K^+^-ATPase and Na^+^-K^+^-ATPase, similar mechanisms have been described, with an ion-occluded conformational E(2) state and an increase in affinity of H^+^ binding in the first case and K^+^ binding in the second. Since the direct binding of clotrimazole to these enzymes has not been shown, the observed effects may be due to its alteration of the membrane structure and dynamics, as has been observed for other molecules such as diethylstilbestrol [26].

We here used DMPC and POPC membranes to study the way in which clotrimazole inserts itself in these membranes and how it affects their structure and dynamics. The DMPC membrane has been widely used to study the effects of intrinsic molecules on membrane properties. DMPC membranes undergo a very cooperative phase transition and the type of perturbation produced by intrinsic molecules is very informative with respect to their interaction with the membrane. Following this technique, we found that clotrimazole induced a decrease in the temperature at the beginning of the phase transition from gel to fluid, with the disappearance of the pretransition at low clotrimazole concentrations. This behaviour is characteristic of the insertion of the intrinsic molecule into the hydrophobic part of the bilayer. It also informs us that at high concentrations of clotrimazole, such as 10:1 (DMPC/clotrimazole molar ratio), a shoulder appears at the higher temperature edge for the main transition, indicating the formation of a phase with a high concentration of clotrimazole due to limited solubility in the DMPC membrane. Fluid immiscibilities have been previously observed for other intrinsic molecules like vitamin K [30,31] curcumin [32] and vitamin E [33].

To study the location of clotrimazole in the bilayer we used 2D-NOESY ^1^H MAS-NMR and a model membrane of POPC. The shifting of the POPC resonances upfield, attributed to the aromatic groups found in the clotrimazole molecule, indicated that the protons bound to the C3 and C2 carbon atoms of the fatty acyl chains of the phospholipid were maximally shifted, which further implied that the highest probability of finding these aromatic groups was near these first carbon atoms of the fatty acyl chains. This probability was also confirmed by the cross-relaxation rates between protons of clotrimazole and POPC, which showed that the maximum proximities of the protons bound to the different groups of clotrimazole those for the protons bound to the C3 and C2 carbons of the fatty acyl chains. It should be remarked that protons bound to the imidazole group seemed to be closer to C2, whereas all the others were closer to C3, suggesting that more polar properties in this imidazole group localizes it slightly nearer the lipid–water interface. This technique has already been widely used to locate small molecules in phospholipid membranes [15] and in our laboratory it has been applied to locate, for example, diethylstilbestrol [26], among others. This technique is very useful because it allows the direct detection of protons bound to the studied molecule and it is not dependent on changes taking place in the bilayer or in the phospholipid molecules. However, it has the disadvantage, which is common to most NMR techniques, of needing high concentrations of the small hydrophobic molecule.

Clotrimazole possesses some structural similarities with other molecules previously studied in our laboratory using the same NMR technique, such as curcumin [27] and diethystilbestrol [26], since all these molecules are hydrophobic and bind to membranes; however, they also exhibit polar groups and are therefore amphipathic. In the three cases using 2D-NOESY ^1^H MAS-NMR and dynamics simulations, we have observed that they preferentially localize in the hydrophobic matrix but relatively near to the polar part of the membrane. This is also the case with another amphipathic molecule, of the same type as the steroid hormone estradiol, that was studied using NMR techniques, and it was observed that the preferred disposition was with the main axis of the molecule parallel to the membrane surface, so that the hydroxyl groups could interact with the lipid–water interface [34].

Some studies on the molecular dynamics of clotrimazole with respect to its interaction with proteins can be found in the literature [35,36,37], but this is the first study that addresses the location of this molecule in a lipid membrane using molecular dynamics. Our experimental results were in very good agreement with those obtained using molecular dynamics simulations, with both approaches suggesting the same location for clotrimazole in the lipid bilayer.

## 5. Conclusions

In this work we used DSC, MAS-NMR and molecular dynamics simulations. DSC showed that clotrimazole disordered and fluidized DMPC membranes and, at high concentrations, formed domains rich in clotrimazole with fluid immiscibilities. NMR and molecular dynamics showed that clotrimazole localizes in the hydrophobic part of the phospholipid bilayer, but not far away from the polar part. In summary, this study may be useful to understand the effect of clotrimazole on SERCA ATPases since its location suggests that it may interfere with the membrane surface, which is where the binding of ions take place. At the same time, knowing the interaction with membranes and the location in the bilayer may be useful when designing nanoparticles for pharmaceutical uses of clotrimazole.

## Figures and Tables

**Table 1:** Changes in ΔH after the addition of clotrimazole to DMPC membranes.

[Clotrimazole]/[DMPC]	Molar Ratio DMPC:Clotrimazole	ΔH (kcal/mol)
0	1:0	6.626 ± 0.040
0.02	50:1	6.548 ± 0.036
0.05	20:1	6.386 ± 0.284
0.1	10:1	6.316 ± 0.182
0.2	5:1	6.342 ± 0.090
0.5	2:1	6.348 ± 0.411

## Supplementary Materials

The following are available online at https://www.mdpi.com/article/10.3390/biom11091304/s1, Figure S1: 1H MAS-NMR spectra of POPC/clotrimazole mixtures.
