# Supplementary material for: Clotrimazole Fluidizes Phospholipid Membranes and Localizes at the Hydrophobic Part near the Polar Part of the Membrane
Source: Biomolecules. 2021 Sep 2;11(9):1304. doi: 10.3390/biom11091304 (PMC8464689; doi:10.3390/biom11091304)
Supplement: Supplementary file 1 [file biomolecules-11-01304-s001.zip › biomolecules-1344808-supplementary.pdf]

Supplementary Materials

Clotrimazole Fluidizes Phospholipid Membranes and Localizes at the Hydrophobic Part near the Polar Part of the Membrane

Alessio Ausili, Illya Yakymenko, José A. Teruel and Juan C. Gómez-Fernández*

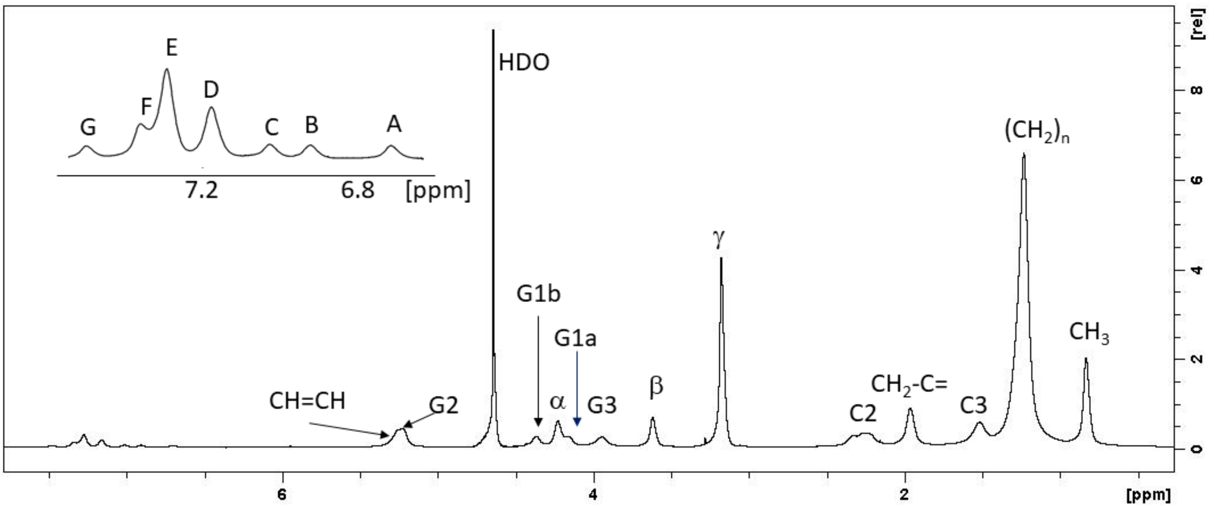

Figure S1. ^{1}H MAS-NMR spectra of POPC/clotrimazole mixtures. The molar ratio was 5:1 phospholipid/clotrimazole and the temperature was 25 °C. The identification of the resonances was undertaken according to the nomenclature for the different carbons used in Figure 1. Note that the symbols A, B, C, D, E, F and G are used for clotrimazole carbons.
